# Mesitylene Tribenzoic Acid as a Linker for Novel Zn/Cd Metal-Organic Frameworks

**DOI:** 10.3390/ma15124247

**Published:** 2022-06-15

**Authors:** Dana Bejan, Ioan-Andrei Dascalu, Sergiu Shova, Alexandru F. Trandabat, Lucian G. Bahrin

**Affiliations:** 1 Intelcentre, Petru Poni Institute of Macromolecular Chemistry, 700487 Iasi, Romania; bejan.dana@icmpp.ro (D.B.); idascalu@icmpp.ro (I.-A.D.); 2 Department of Inorganic Polymers, “Petru Poni” Institute of Macromolecular Chemistry, 700487 Iasi, Romania; shova@icmpp.ro; 3 SC INTELECTRO Iasi SRL, 700029 Iasi, Romania; ftranda@yahoo.com; 4 Department of Electrical Measurements and Materials, Faculty of Electrical Engineering, Technical University Gh. Asachi Iasi, 070050 Iasi, Romania

**Keywords:** metal-organic framework, mesitylene tribenzoic acid, nitrogen sorption

## Abstract

Three new Metal-Organic Frameworks, containing mesitylene tribenzoic acid as a linker and zinc (**1**) or cadmium as metals (**2**,**3**), were synthesized through solvothermal reactions, using DMF/ethanol/water as solvents, at temperatures of 80 °C (structures **1** and **3**) and 120 °C (structure **2**). Following single-crystal X-ray diffraction, it was found that **1** and **3** crystallize in the *P*2_1_/*c* and C2/*c* space groups and form 2D networks, while **2** crystallizes in the *Fdd*2 space group, forming a 3D network. All three frameworks, upon heating, were found to be stable up to 350 °C. N_2_ sorption isotherms revealed that **1** displays a BET area of 906 m^2^/g. Moreover, the porosity of this framework is still present after five cycles of sorption/desorption, with a reduction of 14% of the BET area, down to 784 m^2^/g, after the fifth cycle. The CO_2_ loading capacity of **1** was found to be 2.9 mmol/g at 0 °C.

## 1. Introduction

One of the greatest challenges humanity faces in modern times is represented by climate change. The steady increase in temperature over the last century has led to detrimental effects, such as perturbed weather patterns and ocean currents, an increase in dry areas, and crop failures, to name but a few. Moreover, it is expected that by the end of this century, extreme weather phenomena will increase in frequency [1]. One of the major contributors to climate change is represented by anthropogenic release of greenhouse gases. Burning fossil fuels for transportation alone releases up to 33 Gt of CO_2_ a year into the atmosphere, along with CO, nitrogen, and sulfur oxides, as well as other volatile organic compounds. This value is estimated to be 40% higher than it was in the 19th century [2]. Various ways to tackle this problem have been proposed, amongst which is the use of alternative, cleaner fuels, such as natural gas (comprising up to 95% methane) or hydrogen. Both of these burn cleaner than liquid hydrocarbons and when compared to gasoline, have higher energy densities per unit of mass [3], the downsize being that storing these fuels usually implies the use of high pressure, which in turn leads to increased costs and increased safety risks [4]. Therefore, developing new materials that can be used to efficiently store gaseous fuels at low pressure and ambient temperature is highly desirable. Another way to reduce the quantity of carbon dioxide in the atmosphere is through carbon capture and storage (CCS). CO_2_ can be captured directly from air, or at industrial sites, where it is produced in large concentrations. To capture it, solvent-based methods [5], or solid adsorbent-based methods can be used [6]. Storing can then be done in appropriate geological sites.

One reoccurring factor that can be found in the topics discussed above is the need for efficient solid adsorbents. Amongst the porous materials that are usually considered for such applications, one category, namely Metal-Organic Frameworks (MOFs), stands out. MOFs are porous crystalline materials comprising metal nodes or clusters, bound together by organic linkers, to form one, two, or three-dimensional networks [7,8]. Some of their main advantages over other porous materials, such as activated carbon or zeolites, are their large surface areas and pore volumes, as well as great structural diversity [9], thanks to which MOFs are considered for a wide number of applications in areas such as catalysis [10,11], optoelectronics [12,13], environmental applications [14,15], battery design [16,17], sensor design [18,19], drug delivery [20,21], or gas storage and separation [22,23].

In the past few years, our interest in the field has led to several new Metal-Organic Frameworks, as well as a number of organic linkers that can be used in MOF design [24,25,26,27,28,29,30,31,32,33,34,35,36]. With the goal of obtaining novel MOFs with permanent porosity which have the potential to be used for gas storage, in this work we synthesized and characterized three new Metal-Organic Frameworks, obtained using Zn (II) and Cd (II) as metal sources and 2,4,6-tris(4-carboxyphenyl)-1,3,5-trimethylbenzene (mesitylene tribenzoic acid–H_3_MTB) an organic linker which to this day, has only seen limited use in MOF design.

## 2. Materials and Methods

### 2.1. Chemistry

Zinc nitrate hexahydrate was purchased from Carl Roth, while 96% ethanol was purchased from VWR. All other reagents and solvents were purchased from Sigma Aldrich (St. Louis, MO, USA).

The NMR spectra have been recorded on a Bruker NEO 400 instrument (Bruker BioSpin, Rheinstetten, Germany) operating at 400.1 and 100.6 MHz for ^1^H and ^13^C nuclei. Chemical shifts are reported in δ units (ppm) and were referenced to the internal deuterated solvent (DMSO-*d6* reference at 2.51 ppm (^1^H) and 39.4 (^13^C). IR spectra were recorded on a Shimadzu IRTracer-100 instrument (Shimadzu U.S.A. Manufacturing, Inc., Canby, OR, USA). A STA 449F1 JUPITER (Netzsch, GmbH, Selb, Germany) thermal analyzer from Netzsch was employed for the thermogravimetric (TG) measurements at a heating rate of 5 °C min^−1^ between 30 and 700 °C. The data were processed with the NETZSCH PROTEUS 4.2 software (Netzsch, GmbH, Selb, Germany). X-ray diffraction analysis was performed on a Rigaku Miniflex 600 diffractometer (Rigaku, Tokyo, Japan) using CuKα-emission in the angular range of 5–50° (2θ) with a scanning step of 0.01° and a recording rate of 2°/min. Nitrogen and carbon dioxide sorption experiments (up to 1 bar) for the BET surface area and porosity determination were measured with a Quantachrome NOVA 3200e (Quantachrome GmbH & Co. KG, Odelzhausen, Germany) at 77 K and 273 K, respectively. Elemental analyses (C, H) were conducted using a CE440 Elemental Analyzer (Exeter Analytical, Coventry, UK).

#### 2.1.1. Synthesis of H_3_MTB

To a solution of potassium carbonate (6.55 g, 47 mmol) in water (30 mL), 1,4-dioxane (50 mL), and ethanol (30 mL) were added. The mixture was then degassed by passing N_2_ through it for 15 min. 1,3,5-Triiodo-2,4,6-trimethylbenzene [37] (1.5 g, 3 mmol), 4-carboxyphenylboronic acid (1.94 g, 11.7 mmol), and tetrakis(triphenylphosphine)palladium (0) (0.45 g, 0.39 mmol) were then added and the reaction was heated to 100 °C under nitrogen for 48–72 h, until the color turned black. Next, after cooling to room temperature, the mixture was filtered to remove the black palladium precipitate that formed during the reaction. The filtrate was added to water (400 mL) and acidified to a pH value of around 1 using hydrochloric acid, which led to the formation of an abundant white solid. After filtering and washing with water, the solid was recrystallized from ethanol, yielding colorless needles of H_3_MTB (0.89 g, 61%). ^1^H NMR (400 MHz, DMSO-*d6*): δ= 12.98 (s, 3H, COOH), 8.03 (d, 6H, *J* = 8.3 Hz, H ar), 7.37 (d, 6H, *J* = 8.3 Hz, H ar), 1.62 (s, 9H, CH_3_) ppm. ^13^C NMR (100 MHz, DMSO-*d6*): δ= 167.6, 146.3, 139.2, 132.4, 130.1, 130.0, 129.8, 19.6 ppm.

#### 2.1.2. Synthesis of {[Zn_2_LHCO_2_(DMF)_2_H_2_O]·DMF}_n_ (**1**)

In a 6 mL vial, H_3_MTB (24 mg, 0.05 mmol) was dissolved in a mixture of DMF/ethanol (2 mL/0.5 mL). To this, a solution of zinc nitrate hexahydrate (45 mg, 0.15 mmol) in water (0.5 mL) was added. The reaction vial was capped and heated to 80 °C for 24 h. The crystalline product which formed during this time was then filtered, washed with DMF (3 × 5 mL) and air dried, yielding 35 mg of **1** (78% based on H_3_MTB). IR (ATR): ν (cm^−1^) = 3432 (s), 1553 (m), 1402 (s), 1175 (m), 1099 (w), 1018 (w), 959 (w), 868 (w), 756 (s), 642 (w), 473 (m). Elemental analyses for C_40_H_45_N_3_O_12_Zn_2_ calc. C 53.9%, H 5.1%, N 4.7%, found C 53.8%, H 5.1%, N 4.8%.

#### 2.1.3. Synthesis of {[CdL(DMF)] C_2_H_8_N·H_2_O}_n_ (**2**)

In a 6 mL vial, H_3_MTB (24 mg, 0.05 mmol) was dissolved in a mixture of DMF/ethanol (2 mL/0.5 mL). To this, concentrated hydrochloric acid (37%, 0.1 mL, 1.2 mmol), followed by a solution of cadmium acetate hydrate (38 mg, 0.15 mmol) in water (0.5 mL), were added. The reaction vial was capped and heated to 120 °C for 48 h. The large crystals that formed were then filtered, washed with DMF (3 × 5 mL) and air dried, yielding 23 mg of **2** (63% based on H_3_MTB). IR (ATR): ν (cm^−1^) = 1657 (m), 1587 (m), 1537 (m), 1387 (s), 1173 (w), 1096 (m), 1018 (w), 858 (m), 760 (s), 662 (w), 455 (m). Elemental analyses for C_35_H_38_CdN_2_O_8_ calc. C 57.8%, H 5.3%, N 3.9%, found C 57.7%, H 5.3%, N 4.1%.

#### 2.1.4. Synthesis of {[CdHL(DMF)] DMF}_n_ (**3**)

In a 6 mL vial, H_3_MTB (24 mg, 0.05 mmol) was dissolved in a mixture of DMF/ethanol (2 mL/1 mL). To this, cadmium nitrate tetrahydrate (60 mg, 0.19 mmol), followed by acetic acid (0.16 g, 2.6 mmol) were added and the reaction mixture was homogenized. The reaction vial was then capped and heated to 80 °C for 48 h. The crystals that formed were then filtered, washed with DMF (3 × 5 mL) and air dried, yielding 25 mg of **3** (68% based on H_3_MTB). IR (ATR): ν (cm^−1^) = 1713 (m), 1651 (m), 1607 (m), 1393 (s), 1238 (m), 1177 (w), 1099 (m), 1018 (w), 868 (m), 746 (s), 677 (w), 511 (w), 451 (m). Elemental analyses for C_36_H_36_CdN_2_O_8_ calc. C 58.7%, H 4.9%, N 3.8%, found C 58.5%, H 5.0%, N 3.9%.

#### 2.1.5. Activation of **1** and **3**

After air drying, around 50 mg of the crystalline product were suspended in 5 mL ethanol for 24 h, at room temperature. The solvent was then replaced with fresh ethanol and the suspension was allowed to sit at room temperature for another 24 h. The solid was then filtered, air-dried, and activated under vacuum for 6 h at 150 °C. The activated material was then used in sorption experiments.

### 2.2. X-ray Crystallography

X-ray diffraction data were collected on Oxford-Diffraction XCALIBUR Eos CCD diffractometer using graphite-monochromated Mo-Kα radiation. Single crystals were positioned at 40 mm from the detector and 807, 416, and 282 frames were measured each for 50, 125, and 125 s over 1° scan width for **1**, **2,** and **3**, respectively. The unit cell determination and data integration were carried out using the CrysAlisPro package from Oxford Diffraction [38]. Multi-scan correction for absorption was applied. The structures were solved with the ShelXT program using the intrinsic phasing method and refined by the full-matrix least-squares method on F^2^ with ShelXL [39,40]. Olex2 was used as an interface to the ShelX programs [41]. Non-hydrogen atoms were refined anisotropically. The hydrogen atoms attached to carbon were placed geometrically and refined using a riding model. The hydrogen atoms involved in hydrogen bonding were localized from different Fourier maps accounting for the hybridization of the supporting atoms and the hydrogen bond parameters. The positional parameters of disordered fragments in the crystal of **1** and **3** were refined with necessary imposed restraints on geometry and displacement parameters available in the SHELXL program. The molecular plots were obtained using the Olex2 program. Crystal data and some further details concerning X-ray analysis are given in Table 1, whereas the bond lengths and angles are listed in Tables S1–S6.

## 3. Results and Discussion

The first MOF based on mesitylene tribenzoic acid (H_3_MTB) was reported in 2010 and contained Zn as a metal [42]. Since then, several other structures bearing Zn, Zr, or lanthanides have been obtained [25,43,44,45,46,47,48,49,50], however, to the best of our knowledge, no Cd (II) structures with H_3_MTB as a linker have been presented so far.

**Scheme 1:**
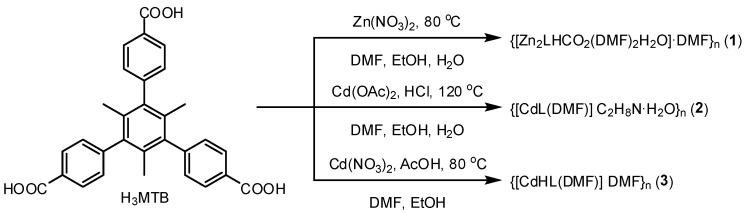
Reaction conditions for the synthesis of the three new MOFs.

Solvothermal reactions of H_3_MTB with zinc (II) nitrate, cadmium (II) acetate, and cadmium (II) nitrate afforded three new metal-organic frameworks, as depicted in Scheme 1. 

**Figure 1:**
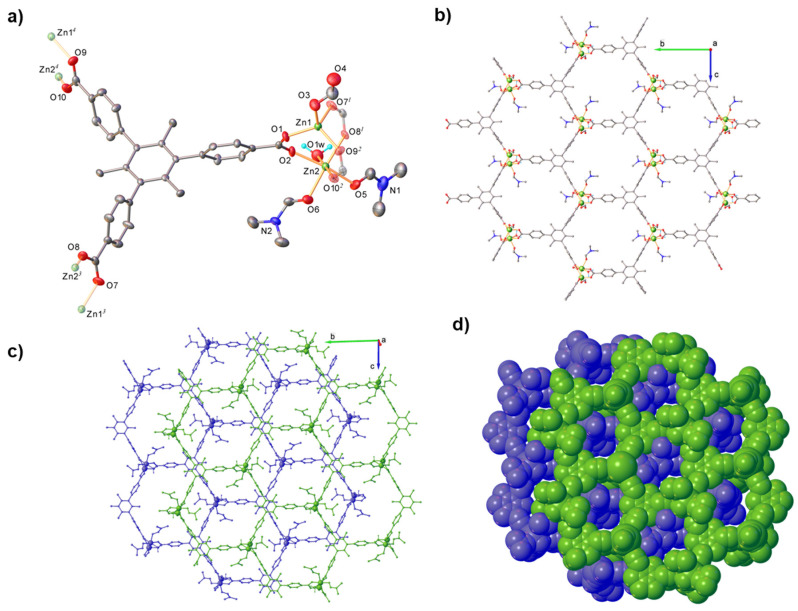
Crystal structure of compound **1**. (**a**) View of the asymmetric unit with thermal ellipsoids at 50% level and selected atom labeling showing the coordination of the Zn atoms. Symmetry-generated atoms are shown with faded colors; (**b**) View of the 2D coordination polymer; (**c**) Crystal packing viewed along the *a*-axis; (**d**) Space-filling model. Non-relevant H-atoms are not shown. Symmetry codes: *^1^*^)^ 1 − *x*, ½ + *y*, ½ − *z*; *^2^*^)^ *x* − 1, *y*, *z* − 1; *^3^*^)^ 1 + *x*, *y*, 1 + *z*; *^4^*^)^ 1 − *x*, ½ + *y*, ½ + *z*.

The crystal structure of the compounds **1**–**3** was determined by a single-crystal X-ray diffraction study. The results of this study for compound **1** are shown in Figure 1. 

According to X-ray crystallography, the charge balance and chemical composition correspond to {[Zn_2_LHCO_2_(DMF)_2_H_2_O]·DMF}_n_ formula. Its structure is described as a neutral two-dimensional coordination polymer, which as an asymmetric unit (Figure 1a) comprises two Zn^2+^ cations, one deprotonated MTB ligand (L^3−^), a formate anion, two DMF and one H_2_O molecule in the first coordination sphere and one co-crystallized DMF molecule. The Zn atoms exhibit different coordination environments. Zn1 is tetrahedrally coordinated by four carboxylate oxygen atoms provided by MTB^3−^ ligands and formate anion, while the Zn2 atom has a distorted octahedral coordination with O6 set of donor atoms provided by two MTB^3−^ carboxylate groups, one water, and two DMF molecules. The separation of two Zn atoms across the bridging carboxylate groups is 3.633(1) Å. The MTB^3−^ anion behaves as a hexadentate ligand bridging six Zn atoms due to *k^2^O,O’* bidentate-bridging coordination function of each carboxylate group. The formate anion fulfills a monodentate coordination function, the second oxygen atom being involved as an acceptor in an intermolecular hydrogen bond towards solvate DMF as a donor of proton. In the crystal, the asymmetric units are self-assembled generating hexagonal two-dimensional coordination polymers, as shown in Figure 1b. The symmetrically related coordination networks in parallel orientation to the 011 plane are interacting through the O-H···O and C-H···O hydrogen bonding formed, respectively, by coordinated water and DMF molecules as donors towards the coordinated oxygen atom of formate anion as acceptor. Their packing occurs in such a way that the centers of the hexagonal openings are overlapped by dinuclear fragments of opposite neighboring layers, as shown in Figure 1c,d. As a result, the free solvent accessible area in the crystal is considerably reduced, constituting only 19.6% of the total unit cell volume.

**Figure 2:**
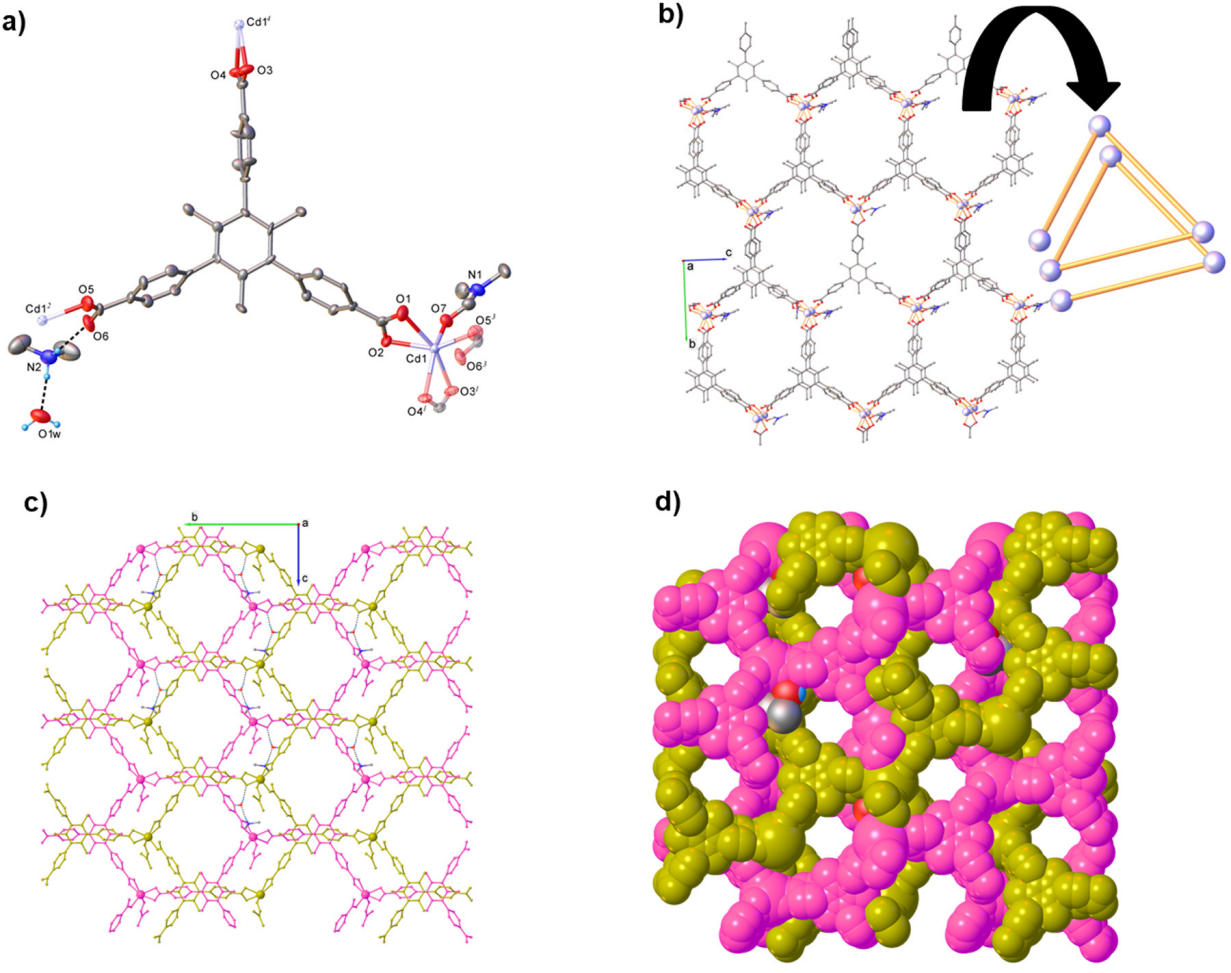
Crystal structure of compound **2**. (**a**) View of the asymmetric unit with thermal ellipsoids at 50% level and selected atom labeling showing the coordination of the Cd atoms. Symmetry-generated atoms are shown with faded colors; (**b**) View of the 3D coordination polymer, helical chain array of Cd atoms along *a*-axis (enclosed); (**c**) Crystal packing viewed along the *a*-axis; (**d**) Space-filling model. Non-relevant H-atoms are not shown. Symmetry codes: *^1^*^)^ 5/4 − *x*, *y* − 1/4, *z* − 1/4; *^2^*^)^ 3/4 − *x*, ¼ + *y*, *z* − 1/4; *^3^*^)^ ¾ − *x*, *y* − 1/4, ¼ + *z*; *^4^*^)^ 5/4 − *x*, ¼ + *y*, ¼ + *z*.

The crystal structure of compound **2** is illustrated in Figure 2. The asymmetric unit comprises one Cd atom, one MTB^3−^ ligand, and one coordinated DMF molecule, which is completed with one dymethylammonium cation and a solvate water molecule (Figure 2a). The Cd atom is coordinated by five oxygen atoms from two bidentate and one monodentate carboxylate groups of MTB^3−^ ligands, while the sixth is provided by a DMF molecule. Assuming that each bidentate carboxylate group occupies only one coordination position, the coordination geometry corresponds to a distorted tetrahedron. Each MTB^3−^ ligand is triply deprotonated, all carboxylate groups being coordinated to Cd atoms. Two of them are coordinated in *k^2^O,O*’ bidentate-bridging mode, while the third one fulfills a monodentate coordination function. The central benzene rings of two MTB^3−^ ligands surrounding each Cd ion are coplanar, while the third is tilted by 22.8(3)°. Due to this particularity, the self-assembling of asymmetric units in the crystal occurs with the formation of an anionic three-dimensional network containing hexagonal openings of 16 × 17 Å, as shown in Figure 2b. Further analysis of the 3D structure has evidenced that the Cd linked by MTB^3−^ ligands form a helical array directed along the *a* crystallographic axis (Figure 2b enclosed). The adjacent subnets are doubly catenated (Figure 2c) and their offsetting at ~10 Å along the *b*-axis leads to the reduction of the solvent-accessible areas in the crystal. Such a packing (Figure 2c,d) is characterized by the presence of channels running parallel to the *a*-axis with accessible voids of ca. 4985 Å^3^ or 29.2% per unit cell as estimated by the Olex2 program.

**Figure 3:**
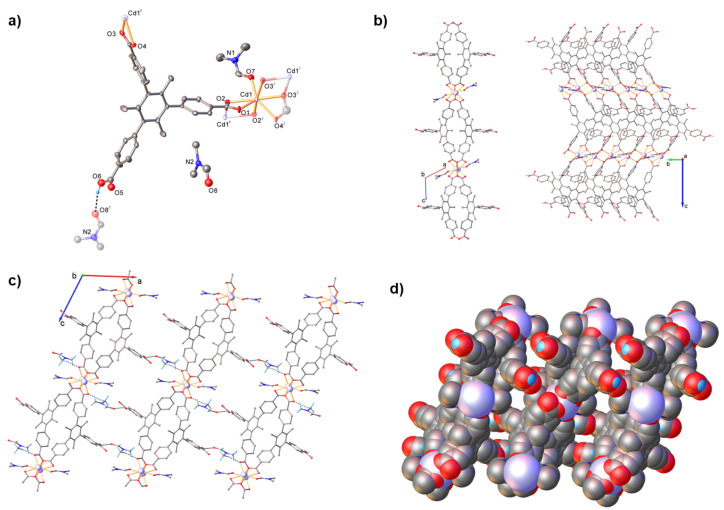
Crystal structure of compound **3**. (**a**) View of the asymmetric unit with thermal ellipsoids at 50% level and selected atom labeling showing the coordination of the Cd atoms. Symmetry-generated atoms are shown with faded colors; (**b**) View of the 2D coordination polymer; (**c**) Crystal packing viewed along the *b* axis; (**d**) Space-filling model. Non-relevant H-atoms are not shown. Symmetry codes: *^1^*^)^ 3/2 − *x*, ½ + *y*, ½ − *z*; ^2)^ *x*, 3 − *y*,1/2 + *z*; ^3)^ 3/2 − *x*, 5/2 − *y*, 1 − *z*; *^4^*^)^ *x*, 3 − *y*, z − 1/2; *^5^*^)^ 1 − *x*, 1 + *y*, ½ + *z*; *^6^*^)^ 3/2 − *x*, 5/2 − *y*, 1 − *z; ^7^*^)^ 3/2 − *x*, 7/2 − *y*, 1 − *z*.

Compound **3** crystallizes in the C2/c space group of the monoclinic system. Its structure is built up from the neutral {CdHL(DMF)}_n_ coordination polymers and co-crystallized DMF molecules in 1:1 ratio. A view of the asymmetric unit is shown in Figure 3a. Each metal ion is coordinated with four carboxylates and a DMF molecule and possesses a highly distorted CdO7 coordination environment. The HMTB^2−^ ligand is doubly deprotonated and links two Cd atoms through two carboxylate groups coordinated in a *μ*^2^-*k*^3^*O,O’:O* tridentate mode. The third non-deprotonated carboxylate is not coordinated, being involved in hydrogen bonding as a donor towards solvate DMF molecule as an acceptor of protons. As a result, the coordination polymer is extending in two directions to form a quite dense two-dimensional coordination network parallel to the 011 plane. A view of the 2D architecture along the b and a-axis is shown in Figure 3b. In the crystal, 2D coordination layers are arranged in parallel packing and the interaction between them occurs through multiple O-H···O and C-H···O hydrogen bonds which involves solvate DMF molecules as donor or acceptor of protons. Consequently, the main crystal packing motif is characterized as a three-dimensional supramolecular network possessing free solvent-accessible voids of 1586 Å3, which constitutes 20.1% of the total unit cell volume.

**Figure 4:**
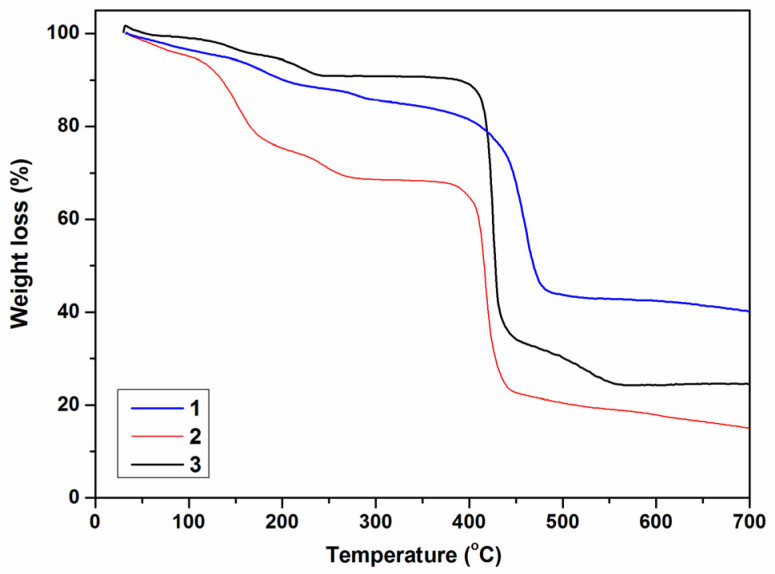
The TGA curves of frameworks **1**, **2,** and **3**.

In order to assess the thermal stability of the coordination networks, thermogravimetric analysis (TGA) was performed on the crystalline compounds **1**, **2,** and **3**. The experimental curves are reported in Figure 4. The results suggest that the initial weight loss which occurs up to 200 °C is associated with the release of water and other solvents. Above 350 °C, the decomposition process of the framework takes place. It is worth pointing out that the MOFs presented here have a relatively high thermal stability, comparable to other Zn/Cd-containing MOFs [51,52].

**Figure 5:**
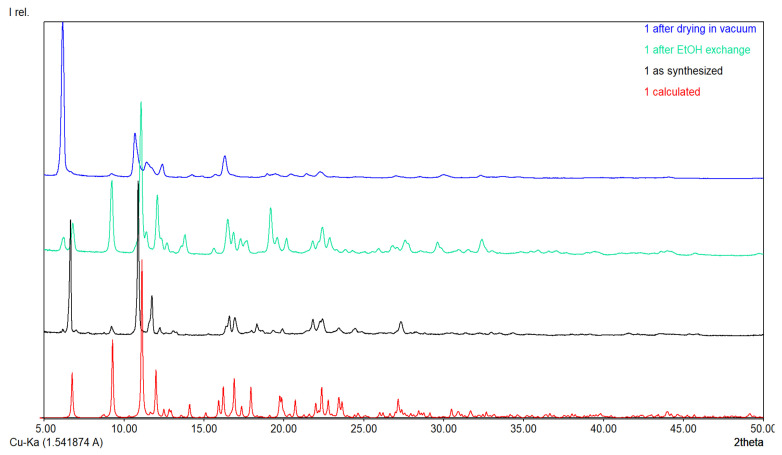
Diffractograms corresponding to compound **1** calculated from the cif file (red), as synthesized (black), after exchange with EtOH (green), and after drying in vacuum (blue).

**Figure 6:**
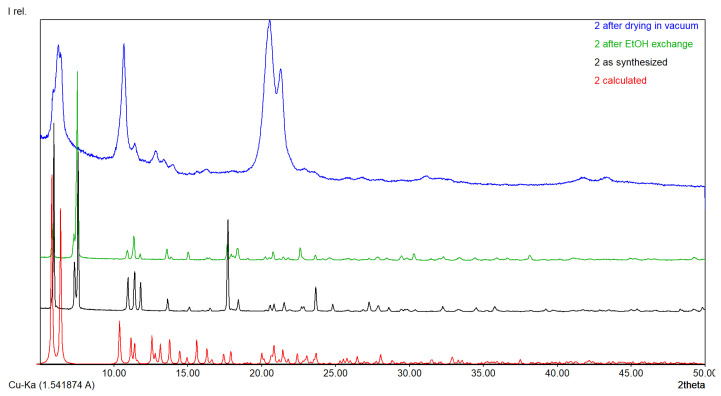
Diffractograms corresponding to compound **2** calculated from the cif file (red), as synthesized (black), after exchange with EtOH (green), and after drying in vacuum (blue).

**Figure 7:**
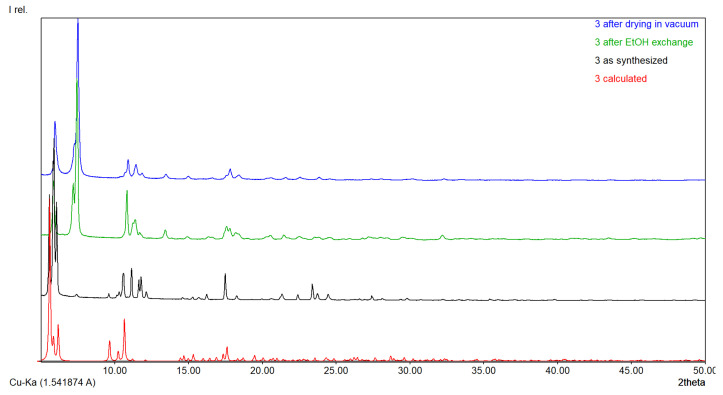
Diffractograms corresponding to compound **3** calculated from the cif file (red), as synthesized (black), after exchange with EtOH (green), and after drying in vacuum (blue).

To evaluate the phase purity of the three new coordination networks, powder X-ray diffractograms were recorded and compared to the simulated spectra (Figure 5, Figure 6 and Figure 7). The phase purity assessment of compounds **1** and **3** as indicated by the similarities between the calculated and experimental diffractograms revealed the presence of a single crystalline phase in each sample. In the case of compound **2**, noticeable differences were observed between the calculated and experimental diffraction patterns (Figure 6). Presumably, this is due to the difference in the temperature at which the data was recorded (180 K for single-crystal X-ray diffraction, which was used to generate the simulated powder diffractogram, and 293 K for the experimental powder X-ray diffractogram), as well as changes in the network which may occur during workup [53].

**Figure 8:**
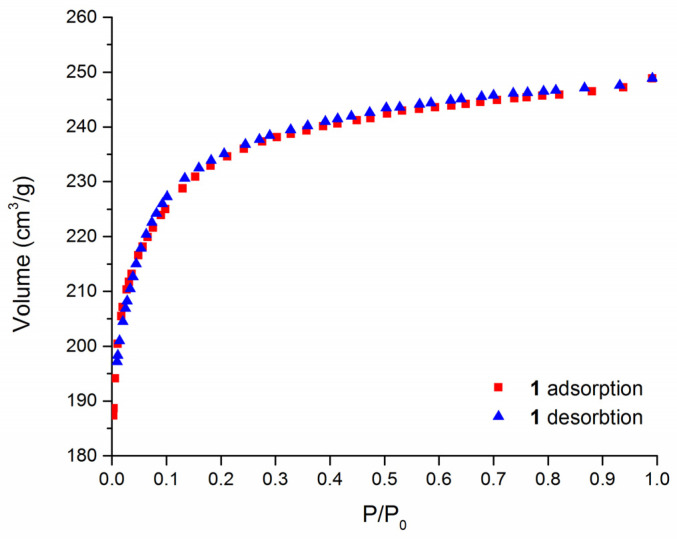
The N_2_ isotherm of **1**, measured at 77 K.

In order to assess their porosity, **1** and **3** were activated prior to gas sorption measurements. The analyzed material was suspended in ethanol for 48 h, followed by filtering, drying, and heating to 150 °C under vacuum, for 6 h. N_2_ isotherms, measured at 77 K are presented in Figure 8 for **1** and Figure S1 for **3**. Both frameworks display type I isotherms, characteristic of microporous materials. While **3** displayed a BET value of only 26 m^2^/g, **1** was found to have a BET value of 906 m^2^/g at *p*/*p*_0_ values situated between 0.01 and 0.05. The values for total pore volume, micropore volume, and mesopore volume for **1** can be found in Table 2, while the pore size distribution of **1**, determined by the BJH method, is presented in Figure S2.

**Figure 9:**
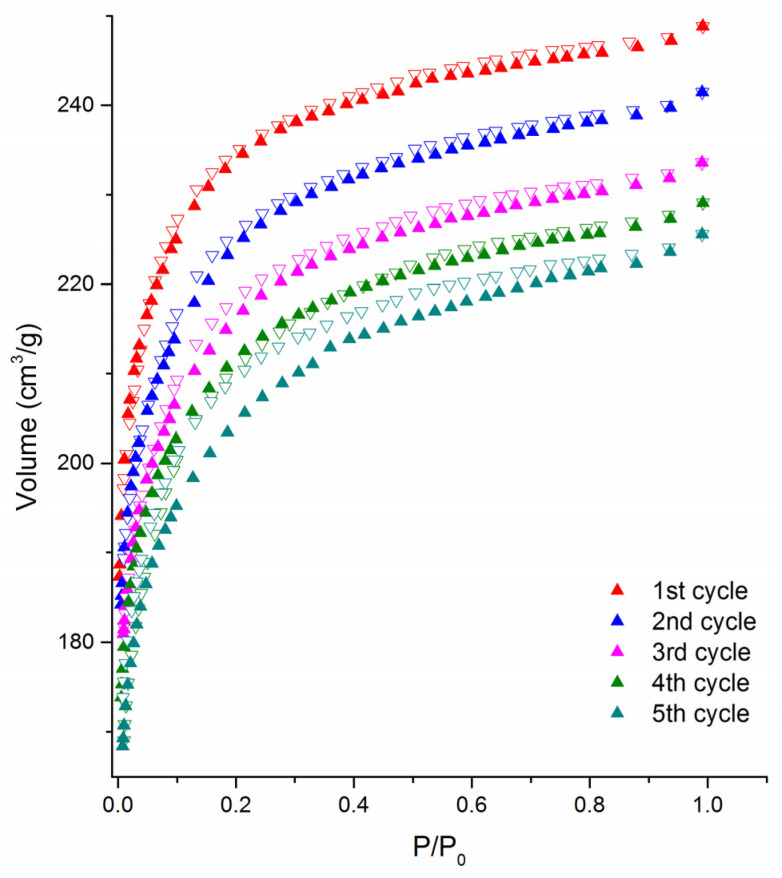
N_2_ isotherms of **1**, where the same sample underwent 5 consecutive cycles of sorption/desorption. Full triangles indicate sorption isotherms and hollow triangles indicate desorption isotherms. BET values were as follows: 906 m^2^/g after cycle 1, 860 m^2^/g after cycle 2, 831 m^2^/g after cycle 3, 815 m^2^/g after cycle 4, and 784 m^2^/g after cycle 5.

The powder X-ray diffractogram registered after the sorption measurement displayed little differences when compared to the one registered for the activated material. Therefore, we decided to submit the same sample to a total of five adsorption/desorption cycles, in order to assess the frameworks’ robustness. As can be seen in Figure 9, **1** displays good sorption properties even after 5 cycles, despite the BET value going down from 906 m^2^/g (cycle 1) to 784 m^2^/g (cycle 5). 

**Figure 10:**
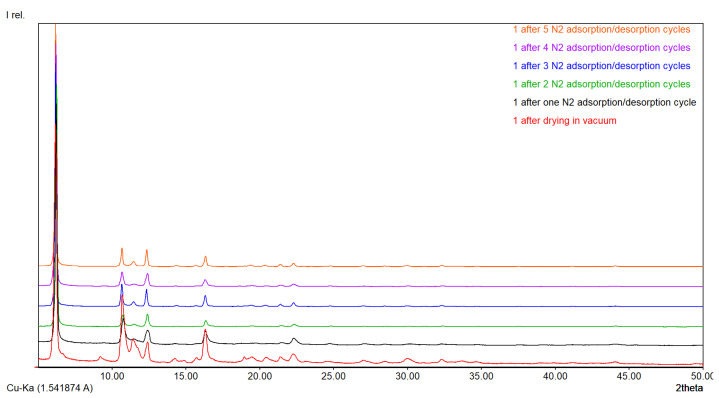
Diffractograms corresponding to compound **1** after drying in vacuum (red) and after 1 (black), 2 (green), 3 (blue), 4 (purple) and 5 (orange) N_2_ adsorption/desorption cycles.

The powder X-ray diffractograms registered after each N_2_ adsorption/desorption cycle, alongside the diffractogram corresponding to framework **1** after activation under heat and vacuum, are presented in Figure 10. We have found that even after five cycles, no major structural modification of the network was observed.

**Figure 11:**
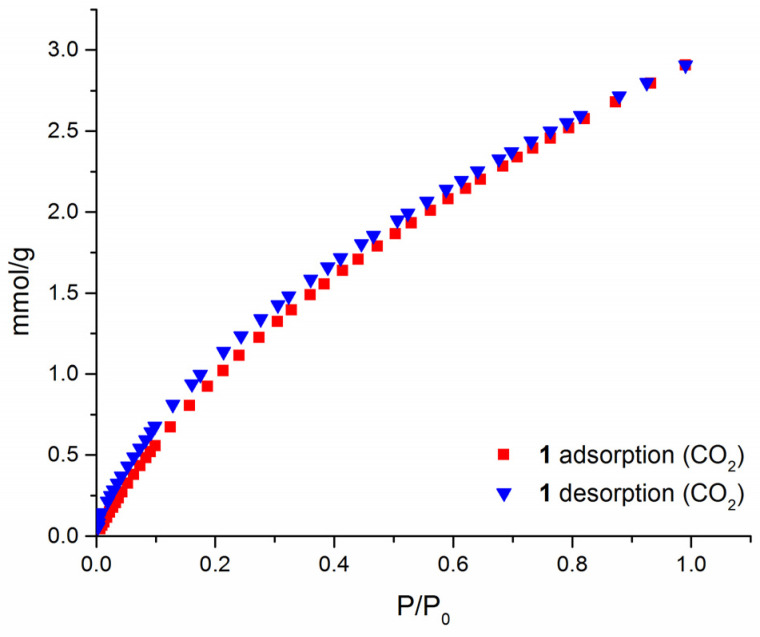
The CO_2_ adsorption/desorption isotherm of **1**, measured at 0 °C.

In the next step, the CO_2_ adsorption properties of framework **1** were investigated. The analysis was performed at 0 °C and the isotherm thus obtained is depicted in Figure 11. It was found that **1** displays a CO_2_ adsorption capacity of 2.9 mmol/g at 0 °C. Moreover, PXRD analysis performed after CO_2_ adsorption/desorption indicated that the network suffers no significant structural modifications (Figure S6).

## 4. Conclusions

To conclude, mesitylene tribenzoic acid was successfully employed as a linker in the solvothermal synthesis of a Zn-containing and two Cd-containing Metal-Organic Frameworks. One of the latter structures proved to be unstable during isolation, as evidenced by PXRD analysis. Out of the remaining frameworks, the Zn-based one was found to display permanent porosity and good N_2_ sorption properties, with a BET value of 906 m^2^/g. Moreover, by subjecting it to five consecutive adsorption/desorption cycles, the framework retained its porous nature, displaying a BET value of 784 m^2^/g after the fifth cycle. When CO_2_ was used as an adsorbate, the same framework was found to have an uptake of 2.9 mmol/g at 0 °C. 

## Figures and Tables

**Table 1:** Crystal data and details of data collection.

	1	2	3
Empirical formula	C_40_H_45_N_3_O_12_Zn_2_	C_35_H_38_CdN_2_O_8_	C_36_H_36_CdN_2_O_8_
*Fw*	890.53	727.07	737.07
Space group	*P*2_1_/*c*	*Fdd*2	*C*2/*c*
*a* [Å]	10.2207(5)	9.0032(5)	34.769(3)
*b* [Å]	28.3147(14)	60.946(2)	7.5285(4)
*c* [Å]	14.9056(9)	31.1632(10)	33.254(3)
α [°]	90	90	90
β [°]	98.678(5)	90	114.833(11)
γ [°]	90	90	90
*V* [Å^3^]	4264.2(4)	17,099.6(12)	7899.7(13)
*Z*	4	16	8
*r*_calcd_ [g cm^−3^]	1.387	1.130	1.239
Crystal size [mm]	0.25 × 0.08 × 0.08	0.15× 0.05 × 0.05	0.25 × 0.02 × 0.02
*T* [K]	200	180	180
*μ* [mm^−1^]	1.187	0.553	0.599
2*Θ* range [°]	3.99 to 50.052	2.936 to 50.052	4.45 to 50.054
Reflections collected	15,409	27,241	16777
Independent reflections	7479[*R*_int_ = 0.0368]	7488[*R*_int_ = 0.0921]	6878[*R*_int_ = 0.0905]
Data/restraints/parameters	7479/82/522	7488/7/423	6878/2/396
*R*_1_^[a]^	0.0890	0.0519	0.0751
*wR*_2_^[b]^	0.2050	0.0641	0.1795
GOF^[c]^	1.071	0.999	1.009
CCDC	2,171,839	2,171,840	2,171,841

**Table 2:** Micropore, mesopore and total pore volume for **1**.

S_BET_ (m^2^/g)	Micropore Volume ^a^ (cm^3^/g)	Mesopore Volume ^b^ (cm^3^/g)	Total Pore Volume (cm^3^/g)
906	0.347	0.021	0.384

^a^ Determined using the t-plot method. ^b^ Determined using the BJH method.

## Data Availability

The CIF files for structures **1** (CCDC No. 2171839), **2** (CCDC No. 2171840) and **3** (CCDC No. 2171841) were uploaded to the Cambridge Crystallographic Data Centre and can be accessed free of charge at https://www.ccdc.cam.ac.uk/structures/.

## Supplementary Materials

The following supporting information can be downloaded at: https://www.mdpi.com/article/10.3390/ma15124247/s1, Figure S1: N_2_ isotherm of **3**.; Figure S2: BJH pore size distribution of **1**; Figures S3–S5: IR spectra for **1**, **2** and **3**; Figure S6: Diffractograms corresponding to framework **1** as synthesized (black), after activation (blue) and after CO_2_ adsorption/desorption (red); Table S1: Selected bond lengths [Å] for 1; Table S2: Selected angles [°] for **1**; Table S3: Selected bond lengths [Å] for **2**; Table S4: Selected angles [°] for **2**; Table S5: Selected bond lengths [Å] for **3**; Table S6: Selected angles [°] for **3**; CIF files of **1**, **2** and **3**.
