# Supplementary material for: Mesitylene Tribenzoic Acid as a Linker for Novel Zn/Cd Metal-Organic Frameworks
Source: Materials (Basel). 2022 Jun 15;15(12):4247. doi: 10.3390/ma15124247 (PMC9227569; doi:10.3390/ma15124247)
Supplement: Supplementary file 1 [file materials-15-04247-s001.zip › materials-1764678-supplementary.pdf]

Supporting Information

for

Mesitylene Tribenzoic Acid as a linker for novel Zn/Cd Metal-Organic Frameworks

Dana Bejan [1,+], Ioan-Andrei Dascalu [1,+], Sergiu Shova [2], Alexandru F. Trandabat [3,4] and Lucian G. Bahrin [1,*]

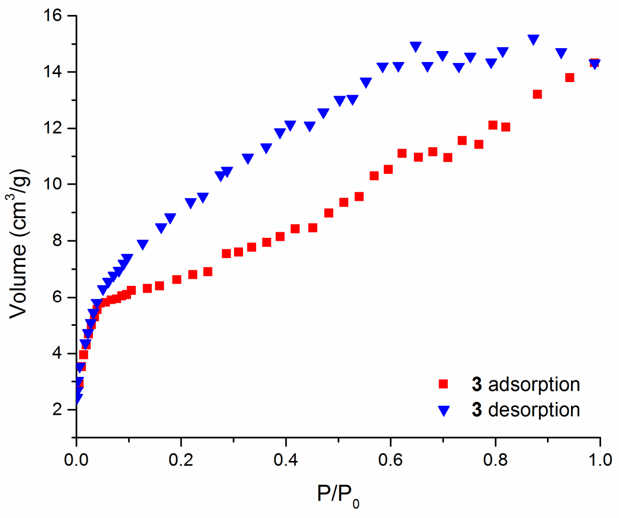

Figure S1. N_2 isotherm of **3**.

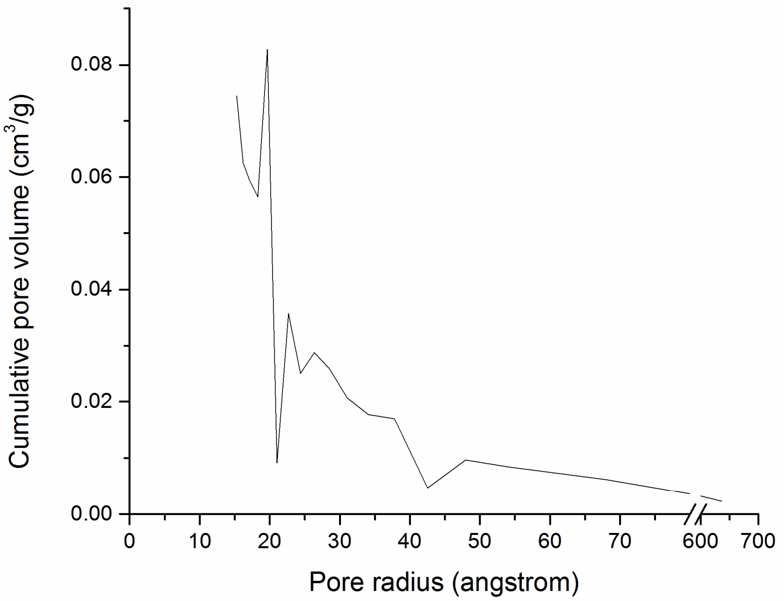

Figure S2. BJH pore size distribution of 1.

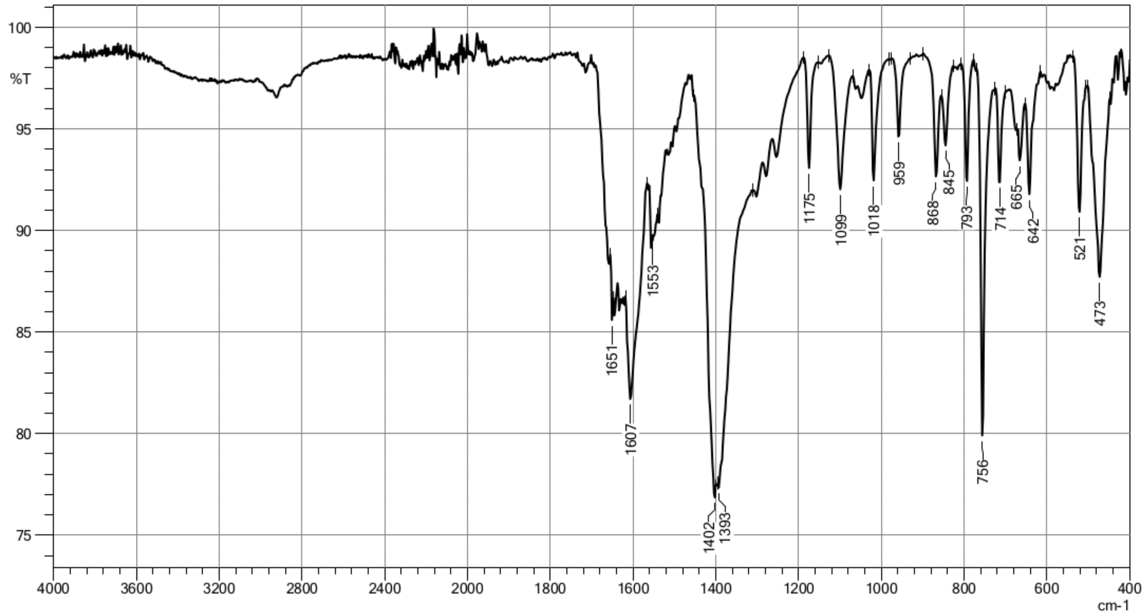

Figure S3. Ir spectrum of **1.**

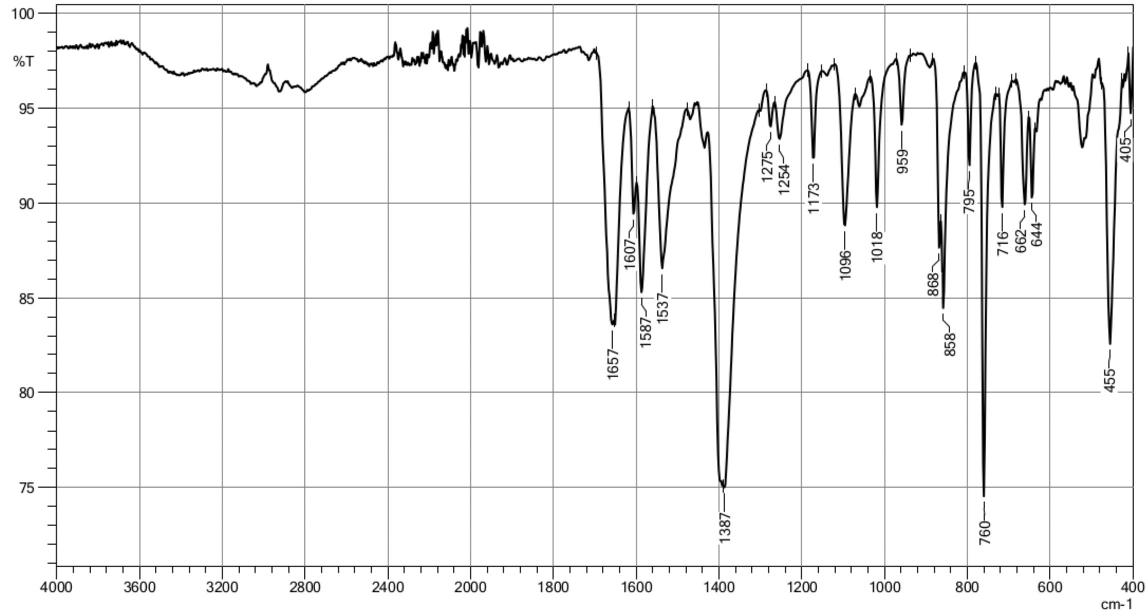

Figure S4. Ir spectrum of **2.**

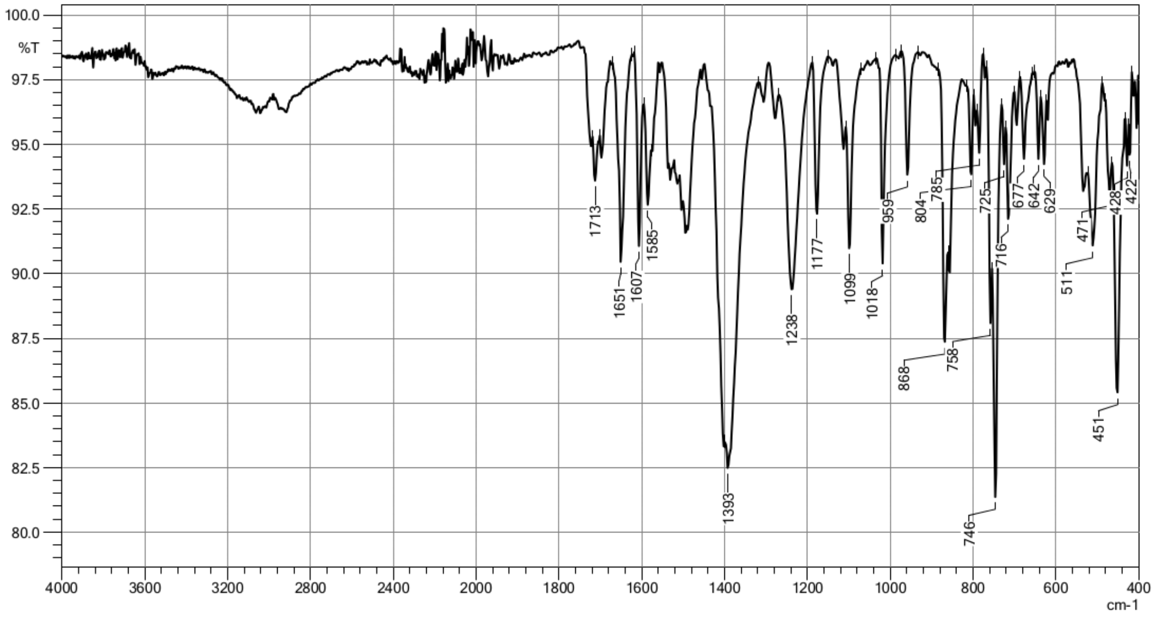

Figure S5. IR spectrum of **3.**

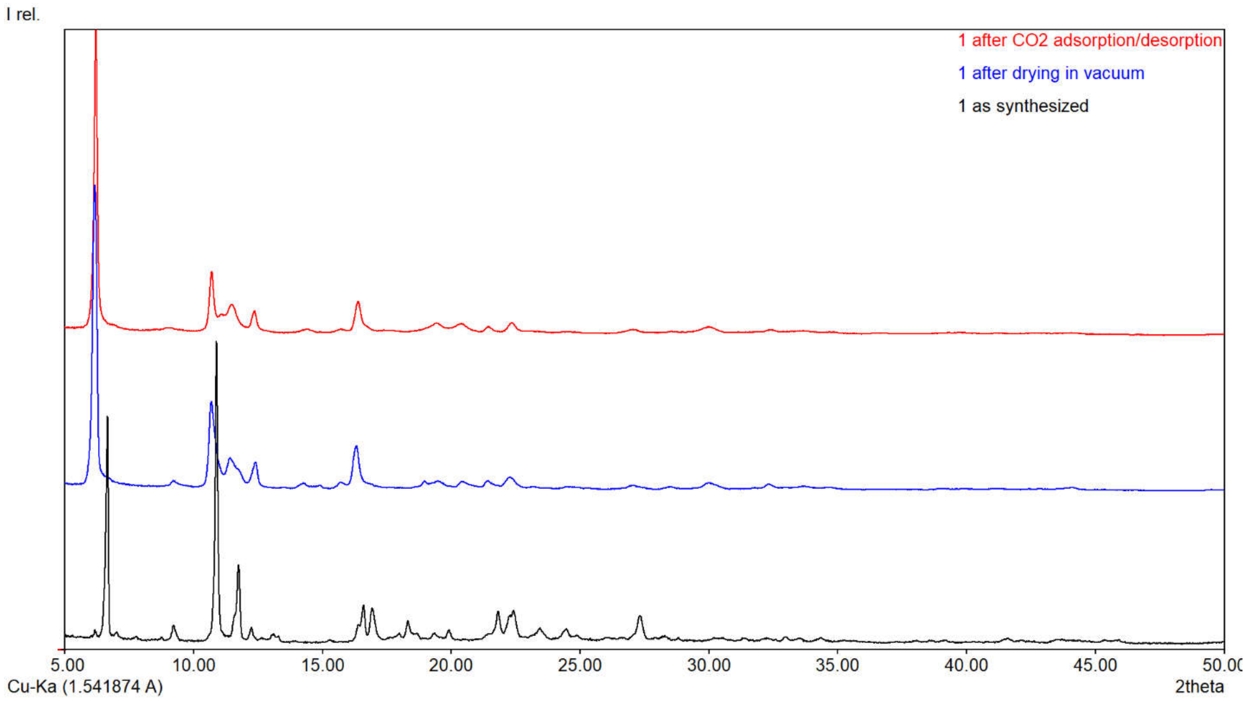

Figure S6. Diffractograms corresponding to framework **1** as synthesized (black), after activation (blue) and after CO_2 adsorption/desorption (red).

Table S1. Selected bond lengths [Å] for **1**

Zn1-O1	1.928(4)	C5-C8	1.497(8)
Zn1-O3	1.982(5)	C6-C7	1.375(9)
Zn1-O7^{1}	1.933(5)	C8-C9	1.401(8)
Zn1-O9^{2}	1.944(5)	C8-C13	1.402(8)
Zn2-O1*w*	2.116(5)	C9-C10	1.401(8)
Zn2-O2	2.086(5)	C9-C15	1.522(8)
Zn2-O6	2.107(5)	C10-C11	1.401(8)
Zn2-O8^{1}	2.083(5)	C10-C24	1.500(8)
Zn2-O10^{2}	2.072(5)	C11-C12	1.413(8)
Zn2-O5	2.088(9)	C11-C16	1.513(8)
O1-C1	1.265(8)	C12-C13	1.413(8)
O2-C1	1.245(8)	C12-C17	1.493(8)
O3-C31	1.220(10)	C13-C14	1.505(8)
O4-C31	1.257(10)	C17-C18	1.388(9)
O6-C35	1.232(9)	C17-C22	1.401(8)
O7-C28	1.249(9)	C18-C19	1.376(9)
O8-C28	1.247(9)	C19-C20	1.386(9)
O9-C23	1.252(9)	C20-C21	1.393(9)

O10-C23	1.248(8)	C20-C23	1.518(9)
N2-C35	1.320(9)	C21-C22	1.373(9)
N2-C36	1.464(10)	C24-C25	1.393(9)
N2-C37	1.469(11)	C24-C30	1.394(9)
C1-C2	1.500(8)	C25-C26	1.380(9)
C2-C3	1.388(9)	C26-C27	1.374(10)
C2-C7	1.396(9)	C27-C28	1.521(9)
C3-C4	1.370(9)	C27-C29	1.352(10)
C4-C5	1.394(8)	C29-C30	1.395(10)
C5-C6	1.392(9)		

[1] 1 – *x*, – ½ + *y*, ½ – *z*; [2] – 1 + *x*, + *y*, – 1 + *z*

Table S2. Selected angles [°] for **1**

O1-Zn1-O3	104.7(2)	C9-C8-C13	120.9(5)
O1-Zn1-O7[1]	113.6(2)	C13-C8-C5	119.3(5)
O1-Zn1-O9[2]	116.4(2)	C8-C9-C15	120.8(5)
O7[1]-Zn1-O3	102.7(2)	C10-C9-C8	119.0(5)
O7[1]-Zn1-O9[2]	115.1(2)	C10-C9-C15	120.3(5)
O9[2]-Zn1-O3	101.8(2)	C9-C10-C11	121.6(6)
O2-Zn2-O1*w*	82.6(2)	C9-C10-C24	119.0(5)
O2-Zn2-O6	87.3(2)	C11-C10-C24	119.4(5)
O2-Zn2-O5	167.6(4)	C10-C11-C12	118.7(5)
O6-Zn2-O1*w*	92.8(2)	C10-C11-C16	119.9(5)
O8[1]-Zn2-O1*w*	86.8(2)	C12-C11-C16	121.4(5)
O8[1]-Zn2-O2	96.30(19)	C11-C12-C17	119.7(5)
O8[1]-Zn2-O6	176.3(2)	C13-C12-C11	120.3(5)
O8[1]-Zn2-O5	91.8(3)	C13-C12-C17	120.0(5)
O10[2]-Zn2-O1*w*	178.1(2)	C8-C13-C12	119.4(5)
O10[2]-Zn2-O2	98.43(19)	C8-C13-C14	121.1(5)
O10[2]-Zn2-O6	85.7(2)	C12-C13-C14	119.5(5)
O10[2]-Zn2-O8[1]	94.60(19)	C18-C17-C12	120.7(5)
O10[2]-Zn2-O5	90.3(4)	C18-C17-C22	117.9(5)
O5-Zn2-O6	97.6(9)	C22-C17-C12	121.4(5)
O5-Zn2-O1*w*	88.4(4)	C19-C18-C17	121.4(6)
C1-O1-Zn1	125.3(4)	C18-C19-C20	120.5(6)
C1-O2-Zn2	142.1(4)	C19-C20-C21	118.7(6)
C31-O3-Zn1	117.4(6)	C19-C20-C23	120.6(6)
C35-O6-Zn2	122.6(5)	C21-C20-C23	120.7(6)
C28-O7-Zn1[3]	122.4(5)	C22-C21-C20	120.7(6)
C28-O8-Zn2[3]	141.8(4)	C21-C22-C17	120.8(6)
C23-O9-Zn1[4]	123.9(4)	O9-C23-C20	116.1(6)

C23-O10-Zn2[4]	140.3(5)	O10-C23-O9	127.4(6)
C35-N2-C36	121.1(7)	O10-C23-C20	116.5(7)
C35-N2-C37	121.6(7)	C25-C24-C10	121.7(6)
C36-N2-C37	117.2(7)	C25-C24-C30	118.4(6)
O1-C1-C2	116.0(6)	C30-C24-C10	119.9(5)
O2-C1-O1	125.6(6)	C26-C25-C24	120.3(6)
O2-C1-C2	118.3(6)	C27-C26-C25	121.0(7)
C3-C2-C1	119.8(6)	C26-C27-C28	120.4(6)
C3-C2-C7	118.3(6)	C29-C27-C26	119.1(6)
C7-C2-C1	121.9(6)	C29-C27-C28	120.5(7)
C4-C3-C2	120.8(6)	O7-C28-C27	116.2(7)
C3-C4-C5	121.5(6)	O8-C28-O7	126.3(6)
C4-C5-C8	120.7(5)	O8-C28-C27	117.5(7)
C6-C5-C4	117.5(5)	C27-C29-C30	121.7(7)
C6-C5-C8	121.8(5)	C24-C30-C29	119.4(6)
C7-C6-C5	121.4(6)	O3-C31-O4	126.1(9)
C6-C7-C2	120.5(6)	O6-C35-N2	124.6(7)
C9-C8-C5	119.8(5)		

[1] 1 – *x*, – ½ + *y*, ½ – z; [2] – 1 + *x*, + *y*, – 1 + z; [3] 1 – *x*, ½ + *y*, ½ – z; [4] 1 + *x*, + *y*, 1 + *z*

Table S3. Selected bond lengths [Å] for **2**

Cd1-O1	2.474(5)	C8-C13	1.419(8)
Cd1-O2	2.252(5)	C9-C10	1.366(9)
Cd1-O3[1]	2.334(5)	C9-C15	1.577(8)
Cd1-O4[1]	2.393(4)	C10-C11	1.416(8)
Cd1-O5[2]	2.166(6)	C10-C17	1.516(9)
Cd1-O7	2.297(5)	C11-C12	1.402(8)
Cd1-C23[1]	2.697(7)	C11-C16	1.541(9)
O1-C1	1.279(8)	C12-C13	1.408(9)
O2-C1	1.219(8)	C12-C24	1.479(9)
O3-C23	1.245(8)	C13-C14	1.501(8)
O4-C23	1.253(9)	C17-C18	1.408(10)
O5-C30	1.243(10)	C17-C22	1.392(10)
O6-C30	1.242(9)	C18-C19	1.333(9)
O7-C31	1.254(8)	C19-C20	1.368(10)
N1-C31	1.301(9)	C20-C21	1.361(9)
N1-C32	1.474(8)	C20-C23	1.522(9)
N1-C33	1.456(9)	C21-C22	1.366(8)
C1-C2	1.492(9)	C24-C25	1.409(11)
C2-C3	1.362(9)	C24-C29	1.388(9)
C2-C7	1.377(8)	C25-C26	1.394(9)

C3-C4	1.359(9)	C26-C27	1.354(9)
C4-C5	1.404(9)	C27-C28	1.378(10)
C5-C6	1.380(9)	C27-C30	1.510(9)
C5-C8	1.499(9)	C28-C29	1.368(9)
C6-C7	1.388(9)	N2-C34	1.420(11)
C8-C9	1.405(9)	N2-C35	1.429(11)

^{1} 5/4 – *x*, – ¼ + y, – ¼ + *z*; ^{2} ¾ – *x*, – ¼ + *y*, ¼ + z

Table S4. Selected angles [°] for **2**

O1-Cd1-C23^{1}	137.6(2)	C8-C9-C15	121.0(6)
O2-Cd1-O1	54.64(17)	C10-C9-C8	121.5(6)
O2-Cd1-O31	110.50(18)	C10-C9-C15	117.4(6)
O2-Cd1-O4^{1}	90.96(17)	C9-C10-C11	119.0(6)
O2-Cd1-O7	89.42(19)	C9-C10-C17	123.3(6)
O2-Cd1-C23^{1}	102.1(2)	C11-C10-C17	117.7(6)
O3^{1}-Cd1-O1	161.81(16)	C10-C11-C16	120.2(6)
O3^{1}-Cd1-O4^{1}	55.13(17)	C12-C11-C10	120.3(6)
O3^{1}-Cd1-C23^{1}	27.4(2)	C12-C11-C16	119.5(6)
O4^{1}-Cd1-O1	110.92(18)	C11-C12-C13	121.0(6)
O4^{1}-Cd1-C23^{1}	27.7(2)	C11-C12-C24	120.7(6)
O5^{2}-Cd1-O1	95.1(2)	C13-C12-C24	118.2(6)
O5^{2}-Cd1-O2	148.5(2)	C8-C13-C14	121.5(6)
O5^{2}-Cd1-O3^{1}	100.8(2)	C12-C13-C8	117.6(6)
O5^{2}-Cd1-O4^{1}	110.1(2)	C12-C13-C14	120.9(6)
O5^{2}-Cd1-O7	87.4(2)	C18-C17-C10	120.9(7)
O5^{2}-Cd1-C23^{1}	107.4(2)	C22-C17-C10	121.3(7)
O7-Cd1-O1	99.64(18)	C22-C17-C18	117.8(6)
O7-Cd1-O3^{1}	89.90(18)	C19-C18-C17	119.6(8)
O7-Cd1-O4^{1}	142.52(18)	C18-C19-C20	123.5(8)
O7-Cd1-C23^{1}	116.4(2)	C19-C20-C23	122.1(7)
C1-O1-Cd1	86.0(4)	C21-C20-C19	117.1(7)
C1-O2-Cd1	97.9(4)	C21-C20-C23	120.7(7)
C23-O3-Cd1^{3}	92.8(5)	C20-C21-C22	122.4(7)
C23-O4-Cd1^{3}	89.8(4)	C21-C22-C17	119.7(7)
C30-O5-Cd1^{4}	112.4(6)	O3-C23-Cd1^{3}	59.8(4)
C31-O7-Cd1	122.7(5)	O3-C23-O4	122.3(7)
C31-N1-C32	122.7(7)	O3-C23-C20	119.2(8)
C31-N1-C33	122.0(7)	O4-C23-Cd1^{3}	62.5(4)

C33-N1-C32	115.3(7)	O4-C23-C20	118.5(7)
O1-C1-C2	118.6(7)	C20-C23-Cd1[3]	178.9(6)
O2-C1-O1	121.3(7)	C25-C24-C12	119.4(7)
O2-C1-C2	120.1(7)	C29-C24-C12	123.4(7)
C3-C2-C1	124.6(7)	C29-C24-C25	117.2(7)
C3-C2-C7	116.7(6)	C26-C25-C24	121.2(7)
C7-C2-C1	118.7(7)	C27-C26-C25	120.9(8)
C4-C3-C2	123.9(7)	C26-C27-C28	117.3(7)
C3-C4-C5	119.2(7)	C26-C27-C30	119.9(8)
C4-C5-C8	120.7(7)	C28-C27-C30	122.8(7)
C6-C5-C4	118.2(7)	C29-C28-C27	124.0(7)
C6-C5-C8	121.1(7)	C28-C29-C24	119.3(8)
C5-C6-C7	120.1(7)	O5-C30-C27	115.4(8)
C2-C7-C6	121.8(7)	O6-C30-O5	125.8(8)
C9-C8-C5	119.9(5)	O6-C30-C27	118.8(8)
C9-C8-C13	120.6(6)	O7-C31-N1	125.6(8)
C13-C8-C5	119.5(6)	C34-N2-C35	115.7(9)

[1] 5/4 – x, – ¼ + *y*, – ¼ + *z*; [2] ¾ – *x*, – ¼ + *y*, ¼ + *z*; [3] 5/4 – x, ¼ + *y*, ¼ + *z*; [4] ¾ – *x*, ¼ + *y*, – ¼ + *z*

Table S5. Selected bond lengths [Å] for **3**

Cd1-O1	2.293(5)	C4-C5	1.380(10)
Cd1-O21	2.339(5)	C5-C6	1.409(10)
Cd1-O2	2.526(5)	C5-C8	1.482(10)
Cd1-O32	2.344(5)	C6-C7	1.403(10)
Cd1-O33	2.385(5)	C8-C9	1.380(10)
Cd1-O42	2.421(5)	C8-C13	1.417(10)
Cd1-O7	2.274(5)	C9-C10	1.399(10)
Cd1-C1	2.748(7)	C9-C15	1.524(9)
Cd1-C23[2]	2.724(8)	C10-C11	1.419(10)
O1-C1	1.254(8)	C10-C17	1.483(10)
O2-C1	1.279(8)	C11-C12	1.401(11)
O3-C23	1.292(8)	C11-C16	1.477(11)
O4-C23	1.239(8)	C12-C13	1.402(10)
O7-C31	1.235(9)	C13-C14	1.496(11)
N1-C31	1.305(10)	C17-C18	1.383(10)
N1-C32	1.451(11)	C17-C22	1.397(10)
N1-C33	1.455(10)	C18-C19	1.399(10)
C1-C2	1.510(10)	C19-C20	1.377(10)
C2-C3	1.392(10)	C20-C21	1.382(11)
C2-C7	1.352(10)	C20-C23	1.496(10)

C3-C4	1.397(10)	C21-C22	1.398(11)

^{1} $^{3}/_{2}$ – *x*, $^{5}/_{2}$ – *y*, 1 – *z*; ^{2} + *x*, 3 – *y*, ½ + *z*; ^{3} $^{3}/_{2}$ – *x*, ½ + *y*, ½ – *z*

Table S6. Selected angles [°] for **3**

O1-Cd1-O2^{1}	106.73(18)	O1-C1-O2	121.3(7)
O1-Cd1-O2	54.28(17)	O1-C1-C2	119.7(7)
O1-Cd1-O3^{2}	86.33(18)	O2-C1-Cd1	66.5(4)
O1-Cd1-O3^{3}	138.91(17)	O2-C1-C2	118.8(7)
O1-Cd1-O4^{3}	94.38(18)	C2-C1-Cd1	164.8(5)
O1-Cd1-C1	26.91(19)	C3-C2-C1	118.0(7)
O1-Cd1-C23^{3}	118.8(2)	C7-C2-C1	121.0(7)
O2^{1}-Cd1-O2	75.0(2)	C7-C2-C3	121.0(7)
O2^{1}-Cd1-O3^{2}	166.81(19)	C2-C3-C4	118.7(7)
O2^{1}-Cd1-O3^{3}	94.59(15)	C5-C4-C3	122.0(7)
O2^{1}-Cd1-O4^{3}	79.00(18)	C4-C5-C6	117.7(7)
O2^{1}-Cd1-C1	93.6(2)	C4-C5-C8	123.2(7)
O2-Cd1-C1	27.67(18)	C6-C5-C8	119.2(7)
O2-Cd1-C23^{3}	152.9(2)	C7-C6-C5	120.3(7)
O2^{1}-Cd1-C23^{3}	83.9(2)	C2-C7-C6	120.2(7)
O3^{2}-Cd1-O2	112.64(15)	C9-C8-C5	121.0(6)
O3^{3}-Cd1-O2	166.28(18)	C9-C8-C13	119.2(7)
O3^{3}-Cd1-O3^{2}	76.0(2)	C13-C8-C5	119.6(7)
O3^{2}-Cd1-O4^{3}	102.25(17)	C8-C9-C10	120.5(6)
O3^{3}-Cd1-O4^{3}	55.14(17)	C8-C9-C15	119.8(6)
O3^{2}-Cd1-C1	97.63(19)	C10-C9-C15	119.6(7)
O3^{3}-Cd1-C1	165.8(2)	C9-C10-C11	120.8(7)
O3^{3}-Cd1-C23^{3}	28.28(18)	C9-C10-C17	120.3(6)
O3^{2}-Cd1-C23^{3}	91.5(2)	C11-C10-C17	118.8(7)
O4^{3}-Cd1-O2	129.23(16)	C10-C11-C16	119.9(8)
O4^{3}-Cd1-C1	115.4(2)	C12-C11-C10	118.6(7)
O4^{3}-Cd1-C23^{3}	27.06(18)	C12-C11-C16	121.5(7)
O7-Cd1-O1	121.93(19)	C11-C12-C13	120.1(7)
O7-Cd1-O2^{1}	89.43(19)	C8-C13-C14	119.3(7)
O7-Cd1-O2	78.91(18)	C12-C13-C8	120.6(7)
O7-Cd1-O3^{2}	81.88(18)	C12-C13-C14	120.0(7)
O7-Cd1-O3^{3}	92.26(18)	C18-C17-C10	123.1(6)
O7-Cd1-O4^{3}	143.68(19)	C18-C17-C22	117.6(7)
O7-Cd1-C1	99.4(2)	C22-C17-C10	119.2(7)
O7-Cd1-C23^{3}	118.2(2)	C17-C18-C19	122.2(7)
C23^{3}-Cd1-C1	142.2(2)	C20-C19-C18	118.8(7)
C1-O1-Cd1	97.2(4)	C19-C20-C21	120.6(7)

Cd1^{1}-O2-Cd1	105.0(2)	C19-C20-C23	119.9(7)
C1-O2-Cd1	85.8(4)	C21-C20-C23	119.5(7)
C1-O2-Cd1^{1}	130.0(4)	C20-C21-C22	119.8(7)
Cd1^{4}-O3-Cd1^{5}	104.0(2)	C17-C22-C21	120.9(7)
C23-O3-Cd1^{5}	119.7(4)	O3-C23-Cd1^{4}	59.3(4)
C23-O3-Cd1^{4}	92.4(4)	O3-C23-C20	116.5(7)
C23-O4-Cd1^{4}	90.2(4)	O4-C23-Cd1^{4}	62.7(4)
C31-O7-Cd1	127.4(5)	O4-C23-O3	121.3(7)
C31-N1-C32	120.8(7)	O4-C23-C20	122.2(7)
C31-N1-C33	122.4(7)	C20-C23-Cd1^{4}	170.1(5)
C32-N1-C33	116.8(7)	O7-C31-N1	126.2(7)
O1-C1-Cd1	55.9(4)		

^{1} 3/2 – *x*, 5/2 – *y*, 1 – *z*; ^{2} 3/2 – *x*, ½ + *y*, ½ – *z*; ^{3} + *x*, 3 – *y*, ½ + *z*; ^{4} + *x*, 3 – *y*, – ½ + *z*; ^{5} 3/2 – *x*, – ½ + *y*, ½ – *z*
